# 2141. Preclinical Evaluation of PA-001: A Novel, Potential Macrocyclic Peptide-Based Treatment for COVID-19 Which Binds to the S2 Subunit of SARS-CoV-2 Spike Protein

**DOI:** 10.1093/ofid/ofad500.1764

**Published:** 2023-11-27

**Authors:** Tatsuro Kawamura, Masaki Ohuchi, Takayuki Nagasawa, Hirofumi Ohashi, Naoko Iwata-Yoshikawa, Nozomi Shiwa-Sudo, Yusuke Sakai, Katsuma Matsui, Masatoshi Matsumoto, Haruaki Kurasaki, Kazutaka Nagatomo, Shoko Ito, Naoki Kawamura, Keiichi Masuya, Noriyo Nagata, Koichi Watashi, Tadaki Suzuki, Hidetomo Kitamura, Masato Murakami

**Affiliations:** PeptiDream Inc., Kawasaki, Kanagawa, Japan; PeptiDream Inc., Kawasaki, Kanagawa, Japan; PeptiDream Inc., Kawasaki, Kanagawa, Japan; Research Center for Drug and Vaccine Development, National Institute of Infectious Diseases, Tokyo, Tokyo, Japan; Department of Pathology, National Institute of Infectious Diseases, Tokyo, Tokyo, Japan; Department of Pathology, National Institute of Infectious Diseases, Tokyo, Tokyo, Japan; Department of Pathology, National Institute of Infectious Diseases, Tokyo, Tokyo, Japan; PeptiDream Inc., Kawasaki, Kanagawa, Japan; PeptiDream Inc., Kawasaki, Kanagawa, Japan; PeptiDream Inc., Kawasaki, Kanagawa, Japan; PeptiDream Inc., Kawasaki, Kanagawa, Japan; PeptiDream Inc., Kawasaki, Kanagawa, Japan; PeptiDream Inc., Kawasaki, Kanagawa, Japan; PeptiAID Inc., Kawasaki, Kanagawa, Japan; Department of Pathology, National Institute of Infectious Diseases, Tokyo, Tokyo, Japan; Research Center for Drug and Vaccine Development, National Institute of Infectious Diseases, Tokyo, Tokyo, Japan; Department of Pathology, National Institute of Infectious Diseases, Tokyo, Tokyo, Japan; PeptiDream Inc., Kawasaki, Kanagawa, Japan; PeptiDream Inc., Kawasaki, Kanagawa, Japan

## Abstract

**Background:**

Despite the approval of a few COVID-19 drugs, various mutations of SARS-CoV-2 continue to emerge and pose as a threat to the efficacy of COVID-19 treatments. To address the unmet clinical need for broad-spectrum treatments, we identified and performed preclinical evaluation of PA-001, a macrocyclic peptide that targets the highly conserved S2 subunit (S2) of the spike protein of SARS-CoV-2, as a potential therapeutic agent with a new mechanism of action.

**Methods:**

A diverse macrocyclic peptide library was constructed and screened for S2 binders employing PeptiDream’s proprietary technology, Peptide Discovery Platform System (PDPS). In vitro antiviral activity was evaluated in VeroE6/TMPRSS2 cells infected with SARS-CoV-2 using RT-qPCR. In vivo efficacy was evaluated in a lethal BALB/c mouse model infected with a mouse-adapted SARS-CoV-2 strain, QHmusX, where PA-001, the remdesivir metabolite GS-441524, alone or in combination, or molnupiravir were therapeutically administered for 3 consecutive days started at 1-day post-inoculation (Fig. 1).

**Figure 1.**
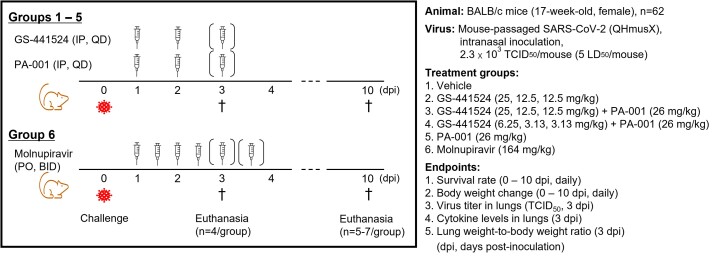
Schematic design of the in vivo efficacy study

**Results:**

PA-001 was identified as a S2 binding peptide through PDPS. PA-001 showed in vitro antiviral activity against wild-type and variant strains of SARS-CoV-2 including Omicron (IC_50_: 1.7 – 9.6 nM). Therapeutic administration of PA-001 completely rescued mice from SARS-CoV-2-caused death at the anticipated clinical dose, while 80% of molnupiravir-administered mice died (Fig. 2A). In addition, treatment with PA-001 alone significantly suppressed body weight loss (Fig. 2B), decreased lung weight-to-body weight ratio (an indicator of lung inflammation), and reduced inflammatory cytokines secretion including IL-6 in lungs, and these effects were enhanced when combined with GS-441524.

**Figure 2.**
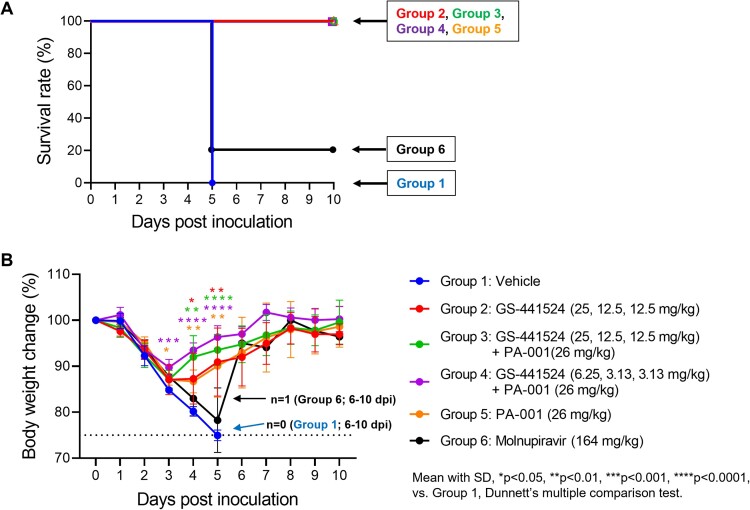
Survival rate and body weight change of SARS-CoV-2-infected mice

**Conclusion:**

The S2-targeting peptide PA-001 showed potent in vitro antiviral activity and in vivo preclinical therapeutic efficacy. These data support the possibility that PA-001 could become a novel drug with a unique mechanism of action for the treatment of COVID-19. Currently, IND submission for PA-001 is in preparation to initiate clinical trials.

**Disclosures:**

**All Authors**: No reported disclosures

